# Shp2–Mitogen-Activated Protein Kinase Signaling Drives Proliferation during Zebrafish Embryo Caudal Fin Fold Regeneration

**DOI:** 10.1128/MCB.00515-17

**Published:** 2018-01-29

**Authors:** Alexander James Hale, Jeroen den Hertog

**Affiliations:** a Hubrecht Institute-KNAW and University Medical Center Utrecht, Utrecht, the Netherlands; b Institute Biology Leiden, Leiden University, Leiden, the Netherlands

**Keywords:** MAPK signaling, Shp2, protein-tyrosine phosphatase, regeneration, zebrafish

## Abstract

Regeneration of the zebrafish caudal fin following amputation occurs through wound healing, followed by formation of a blastema, which produces cells to replace the lost tissue in the final phase of regenerative outgrowth. We show that *ptpn11a^−/−^ ptpn11b^−/−^* zebrafish embryos, lacking functional Shp2, fail to regenerate their caudal fin folds. Rescue experiments indicated that Shp2a has a functional signaling role, requiring its catalytic activity and SH2 domains but not the two C-terminal tyrosine phosphorylation sites. Surprisingly, expression of Shp2a variants with increased and reduced catalytic activity, respectively, rescued caudal fin fold regeneration to similar extents. Expression of *mmp9* and *junbb*, indicative of formation of the wound epidermis and distal blastema, respectively, suggested that these processes occurred in *ptpn11a^−/−^ ptpn11b^−/−^* zebrafish embryos. However, cell proliferation and MAPK phosphorylation were reduced. Pharmacological inhibition of MEK1 in wild-type zebrafish embryos phenocopied loss of Shp2. Our results suggest an essential role for Shp2a–mitogen-activated protein kinase (MAPK) signaling in promoting cell proliferation during zebrafish embryo caudal fin fold regeneration.

## INTRODUCTION

In a process termed epimorphic regeneration, zebrafish (Danio rerio) fully regenerate their heart, retina, spinal cord, and caudal fin after injury (1, 2). Regeneration of the zebrafish caudal fin proceeds sequentially through three distinct phases: wound healing, blastema formation, and regenerative outgrowth. The mechanism of zebrafish embryonic caudal fin fold regeneration is highly similar to adult zebrafish caudal fin regeneration, though it reaches completion within 4 days instead of within 2 weeks (3). Following injury, nearby cells migrate to cover the wound and form an apical epidermal cap that is essential to initiate blastema formation and regenerative outgrowth (4). Many genes have been implicated in the regenerative process (5), and multiple signaling pathways have been validated to be essential for regeneration, including fibroblast growth factor (FGF), sonic hedgehog, bone morphogenetic protein, Wnt, and Notch (6).

Protein tyrosine phosphorylation is at the core of many essential signaling cascades, and the balance between the activities of protein tyrosine kinases and protein tyrosine phosphatases (PTPs) allows an appropriate cellular response to stimuli (7). Src homology 2 domain-containing phosphatase 2 (SHP2) is a cytoplasmic PTP, encoded by the *PTPN11* gene. SHP2 is involved in signaling initiated by various growth factors (8, 9) and has a role downstream in stimulating mitogen-activated protein kinase (MAPK) signaling (10, 11). SHP2 has been implicated as playing a role in a plethora of cellular processes, including proliferation (12, 13), cell migration (14–16), stem cell self-renewal and differentiation (17–20), adipogenesis (21, 22), and hematopoiesis (17, 23). SHP2 and MAPK signaling are indispensable for proper embryonic development (24–28), and SHP2 is mutated in a variety of human diseases (29).

The zebrafish genome contains two *ptpn11* genes, *ptpn11a* and *ptpn11b*, encoding Shp2a and Shp2b, respectively. Both Shp2a and Shp2b are highly homologous to human SHP2 and harbor catalytic activity. Shp2b is dispensable, but Shp2a is not, which is due to differential expression of *ptpn11a* and *ptpn11b* during early development. *ptpn11a^+/+^ ptpn11b^−/−^* and *ptpn11a^+/−^ ptpn11b^−/−^* zebrafish are viable and fertile, yet homozygous *ptpn11a^−/−^ ptpn11b^−/−^* double-knockout zebrafish are embryonically lethal from 5 to 7 days postfertilization (dpf) onward (26).

The SHP2 protein consists of two SH2 domains, followed by a catalytic PTP domain and a C-terminal domain (30). SHP2, like all classical PTPs, mediates dephosphorylation of its substrates through a mechanism involving a catalytic cysteine (C460 in zebrafish Shp2a) and an assisting arginine (R466 in zebrafish Shp2a) in the PTP domain, and mutation of either of these residues abolishes catalytic activity (16, 31–34). The crystal structure of SHP2 shows a closed conformation, with the N-terminal SH2 domain interacting with residues close to the catalytic pocket, thus blocking access of substrates to the catalytic site and impairing catalytic activity (30). Activation of SHP2 is facilitated by dissociation of the SH2 domains from the PTP domain, engendering an open conformation, which allows access of target substrates to the catalytic site (35). Mutation of key residues, such as D61 in the N-terminal SH2 domain, which was identified as causing Noonan syndrome (NS), results in an open conformation of SHP2 and increased catalytic activity (36–38). In Noonan syndrome with multiple lentigenes (NS-ML), mutations were identified close to the catalytic cysteine, such as A461 (A462 in zebrafish Shp2a), which result in strongly reduced activity (38–40).

Importantly, the SH2 domains and C-terminal domain of SHP2 are required for the function of SHP2 in response to growth factor stimulation. The SH2 domains bind to phosphotyrosine-containing target proteins (25). The C-terminal domain mediates interactions with other proteins. Two tyrosines (Y542 and Y580) are particularly important, because when phosphorylated, they constitute binding sites for SH2 domain-containing proteins (41–43), which mediate MAPK activation in response to growth factors (44). Collectively, the studies on the function of the domains of SHP2 show that both the SH2 and C-terminal domain potentiate, but are not definitively required for the stimulation of, MAPK signaling by the PTP domain of SHP2.

Regeneration requires cell survival, migration, proliferation, and differentiation for effective wound healing and replacement of lost tissue (4, 45, 46). MAPK activation following injury is associated with regenerative competence across species (47, 48). The need for MAPK signaling in zebrafish caudal fin regeneration has also been implied (49–51). However, not only MAPK but also phosphoinositide 3-kinase (PI3K), phospholipase Cγ (PLCγ), and signal transducer and activator of transcription (STAT) signaling is activated (52), complicating the conclusion that MAPK signaling is required. Whereas PI3K signaling is essential for zebrafish caudal fin regeneration (45, 53), the evidence supporting a role for MAPK signaling is inconclusive. Hence, the role of SHP2 and MAPK signaling in zebrafish caudal fin regeneration remains to be determined definitively.

We investigated the role of Shp2 in zebrafish embryo caudal fin fold regeneration using homozygous *ptpn11a^−/−^ ptpn11b^−/−^* zebrafish embryos, which lack functional Shp2 (26), and found that Shp2 is required for normal caudal fin fold regeneration. Rescue experiments with mutant Shp2a indicated that functional SH2 domains and catalytic activity were required for its capacity to rescue regeneration, whereas the two tyrosine residues in the C terminus of Shp2a were dispensable. Characterization of the regeneration defect in *ptpn11a^−/−^ ptpn11b^−/−^* zebrafish embryos suggested that formation of the wound epidermis and distal blastema occurred similarly to that in their siblings but that cell proliferation and MAPK phosphorylation were significantly reduced during regenerative outgrowth. In a similar manner, pharmacological inhibition of MEK1, upstream of MAPK, in wild-type zebrafish embryos inhibited regeneration and reduced proliferation during regenerative outgrowth. Collectively, our results demonstrate that Shp2a requires its SH2 domains and catalytic activity for its function in zebrafish embryo caudal fin fold regeneration and likely acts to activate MAPK signaling, which is required to stimulate proliferation during regeneration.

## RESULTS

### Shp2a requires its SH2 domains and catalytic activity for its function in regeneration of the zebrafish embryo caudal fin fold.

**FIG 1:**
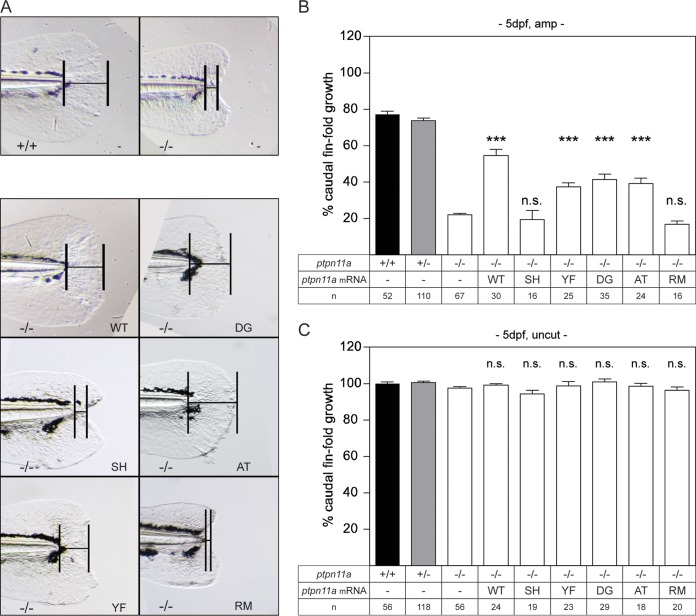
Functional Shp2a is required for regeneration. Zebrafish embryos from a *ptpn11a^+/−^ ptpn11b^−/−^* incross were microinjected at the one-cell stage with synthetic mRNA encoding wild-type Shp2a, SH2 domain mutant Shp2a-R32M-R138M, C-terminal tyrosine mutant Shp2a-Y542F-Y580F, Shp2a-D61G, Shp2a-A462T, or Shp2a-R466M or were not injected (−). At 2 dpf, the caudal fin fold was amputated, and regeneration was assessed at 3 dpa (i.e., 5 dpf and 3 dpa); equivalent uncut controls were included (i.e., 5 dpf, uncut). All the embryos were genotyped. (A) Representative images of amputated *ptpn11a^−/−^ ptpn11b^−/−^* embryo caudal fin folds at 3 dpa. A *ptpn11a^+/+^ ptpn11b^−/−^* sibling in which regeneration of the caudal fin fold was 80% complete by 3 dpa is shown for comparison (top left). (B and C) Regeneration was quantified by measuring the distance from the tip of the notochord to the edge of the caudal fin fold, as indicated (bars in panel A). The means of caudal fin fold growth are depicted relative to caudal fin fold growth of uncut *ptpn11a^+/+^ ptpn11b^−/−^* controls. Means of microinjected amputated (amp) (B) or uncut (C) *ptpn11a^−/−^ ptpn11b^−/−^* embryos were compared to those of noninjected amputated or uncut *ptpn11a^−/−^ ptpn11b^−/−^* embryos. The data were pooled from multiple experiments. Statistical evaluation was performed using a Mann-Whitney U test for comparison of *ptpn11a^−/−^ ptpn11b^−/−^* zebrafish embryos with siblings within amputated or uncut groups, not between amputated and uncut groups. The error bars indicate standard errors of the mean. ***, *P* < 0.001; n.s. not significant.

We have previously shown that homozygous *ptpn11a^−/−^ ptpn11b^−/−^* zebrafish embryos, lacking functional Shp2, fail to regenerate their caudal fin fold following amputation, demonstrating that Shp2a is required for zebrafish caudal fin fold regeneration (54). To validate that impaired regeneration is indeed due to the lack of functional Shp2, we performed rescue experiments using wild-type Shp2a (WT). Next, we determined which signaling domains of Shp2a are required for its function, using SH2 domain or C-terminal domain mutants of Shp2a. To this end, zebrafish embryos from a *ptpn11a^+/−^ ptpn11b^−/−^* incross were microinjected at the one-cell stage with synthetic mRNA encoding wild-type Shp2a; Shp2a-R32M-R138M (SH), in which the essential arginine residues in both SH2 domains were mutated; or Shp2a-Y542F-Y580F (YF), which lacks the two tyrosine phosphorylation sites that are important for signaling. The mRNAs encoding (mutant) Shp2a proteins also encode enhanced green fluorescent protein (eGFP) linked by a peptide-2a cleavage sequence (55). At 2 dpf, eGFP-positive zebrafish embryos were selected, their caudal fin folds were amputated (referred to here as “amputated zebrafish embryos”), and they were allowed to regenerate for 3 days, which results in ∼80% complete regeneration in wild-type zebrafish embryos. Representative photographs of regenerated caudal fin folds of *ptpn11a^−/−^ ptpn11b^−/−^* zebrafish embryos expressing (mutant) Shp2a protein at 3 days postamputation (dpa) are shown in Fig. 1A. Caudal fin fold lengths were determined and are presented as percent caudal fin fold growth, normalized to that of uncut control *ptpn11a^+/+^ ptpn11b^−/−^* zebrafish embryos (Fig. 1B and C). All the zebrafish embryos were subsequently genotyped.

Expression of wild-type Shp2a or the tyrosine phosphorylation site mutant (YF) resulted in significant rescue (*P* < 0.001) of regeneration in *ptpn11a^−/−^ ptpn11b^−/−^* zebrafish embryos. In contrast, expression of the SH2 domain mutant of Shp2a (SH) was unable to rescue regeneration (Fig. 1A and B). Rescue of regeneration did not reach the 80% normally exhibited by control *ptpn11a^−/−^ ptpn11b^−/−^* zebrafish embryos. This was probably due to a combination of mosaicism that occurs when using mRNA injections, resulting in a fraction of the cells not expressing the Shp2a protein, and mRNA injections being transient (56). These results demonstrate that the SH2 domains, but not the C-terminal tyrosine phosphorylation sites, are required for Shp2a function in zebrafish embryo caudal fin fold regeneration.

Next, we tested the rescue capacity of Shp2a mutants with altered catalytic activity. We have previously shown that zebrafish Shp2a mutants with an NS-associated mutation, D61G, or an NS-ML mutation, A462T, have increased or reduced activity, respectively, when tested *in vitro* (57). In addition, we used a Shp2a mutant with a mutation of the conserved arginine (R466M), which lacks catalytic activity, rather than the catalytic cysteine mutant (C460S in zebrafish Shp2a) because Shp2a-C460S may trap substrates (31, 58) and thus have inadvertent dominant effects. Interestingly, the amputated caudal fin folds of *ptpn11a^−/−^ ptpn11b^−/−^* zebrafish embryos expressing Shp2a-D61G (DG) or Shp2a-A462T (AT) but not Shp2a-R466M (RM) regenerated to similar extents (Fig. 1A and B). Small differences were observed between the different Shp2a mutants that rescued caudal fin fold regeneration in the *ptpn11a^−/−^ ptpn11b^−/−^* zebrafish embryos (Fig. 1B). However, using a Mann-Whitney U test with a *post hoc* Monte-Carlo exact test, we established that they were not statistically significant. The fin folds of uncut controls expressing any of the Shp2 mutants were not significantly affected and showed normal growth of the caudal fin fold, with lengths comparable to those in noninjected zebrafish embryos and siblings (Fig. 1C).

**FIG 2:**
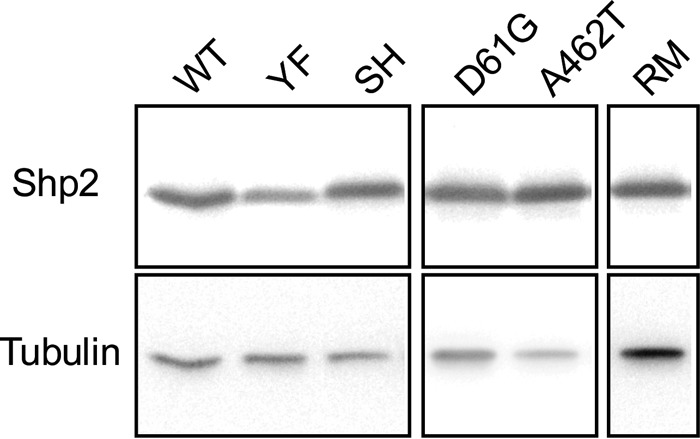
Similar expression levels of Shp2a mutants. Human embryonic kidney 293 T cells were transfected with cytomegalovirus (CMV) promoter-driven expression vectors for zebrafish wild-type Shp2a and Shp2 mutants: Shp2a-Y542F-Y580F, Shp2a-R32M-R138M, Shp2a-D61G, Shp2a-A462T, and Shp2a-R466M. The cells were lysed, and the lysates were run on an SDS-PAGE gel. The blots were probed using a SHP2-specific antibody and developed using enhanced chemiluminescence. The blots were stripped and reprobed for tubulin as a loading control. All the samples were loaded on the same blot.

A trivial explanation for the inability of the catalytically inactive mutant of Shp2a (RM) or the SH2 domain mutant of Shp2a to rescue caudal fin fold regeneration might be that these proteins have reduced stability. Due to low expression levels of (mutant) Shp2a proteins in zebrafish embryos, it was not possible to monitor protein expression *in vivo*. However, transfection of constructs encoding wild-type Shp2a, Shp2a-R32M-R138M, Shp2a-Y542F-Y580F, Shp2a-D61G, Shp2a-A462T, or Shp2a-R466M proteins in HEK293T cells revealed that all Shp2a proteins are expressed to similar extents, albeit expression levels vary from mutant to mutant (Fig. 2). This suggests that the inability of the SH and RM mutants to rescue caudal fin fold regeneration is not due to greatly reduced protein expression or stability, but rather to functional differences in Shp2a function.

Taken together, these results demonstrate that functional Shp2 is required for zebrafish embryo caudal fin fold regeneration. Although the two tyrosine phosphorylation sites in the C-terminal domain are dispensable, the catalytic activity, as well as the SH2 domains, of Shp2 is required for the function of Shp2a in zebrafish embryo caudal fin fold regeneration. Furthermore, the level of catalytic activity harbored by Shp2a appears not to affect the extent of zebrafish embryo caudal fin fold regeneration.

### Markers for formation of the wound epidermis and distal blastema suggest the initial response to amputation occurs normally in zebrafish embryos deficient for Shp2.

**FIG 3:**
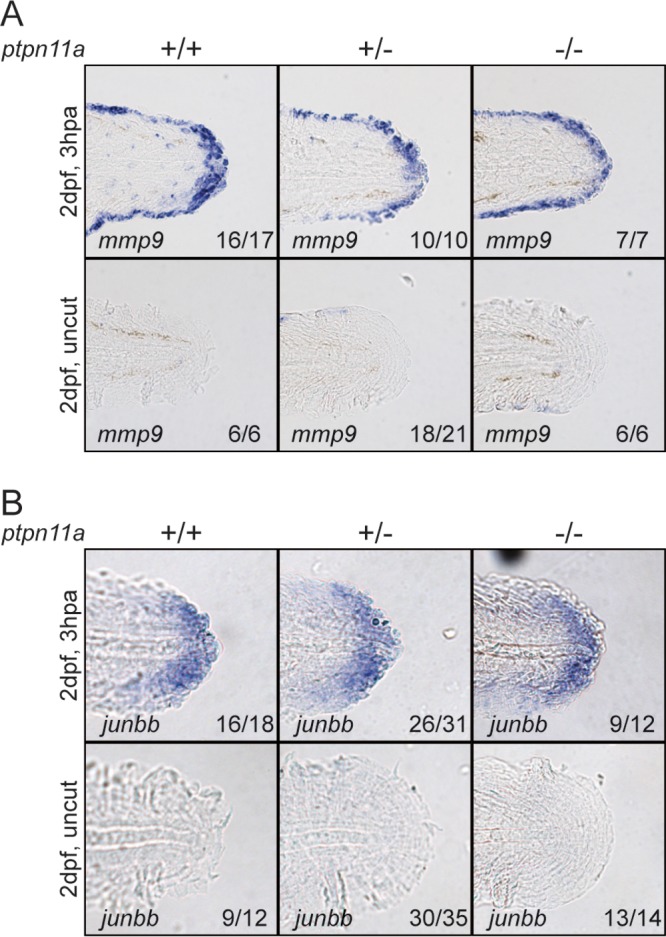
Formation of wound epidermis and distal blastema in zebrafish embryos lacking functional Shp2. At 2 dpf, the caudal fin folds of zebrafish embryos from a *ptpn11a^+/−^ ptpn11b^−/−^* incross were amputated and allowed to regenerate. The embryos were fixed at 3 hpa, or the equivalent for uncut controls, and subjected to hybridization using probes specific for *mmp9* (A) or *junbb* (B). Representative images of caudal fin folds of genotyped embryos are shown, and the number of embryos showing similar patterns/total number of embryos analyzed are indicated in the bottom right corner of each image.

Following amputation, wound healing occurs, and an apical epidermal cap is produced that signals for the formation of the blastema. Thus, successful blastema formation is indicative of successful wound healing (1, 2). Zebrafish embryos from a *ptpn11a^+/−^ ptpn11b^−/−^* incross were fixed at 3 h postamputation (hpa) and subjected to *in situ* hybridization using probes specific for *mmp9* and *junbb*, which are normally upregulated following caudal fin fold amputation (59, 60) and mark the wound epidermis and distal blastema, respectively. All the zebrafish embryos were subsequently genotyped. Expression of *mmp9* (Fig. 3A) and *junbb* (Fig. 3B) was clearly induced in amputated caudal fin folds but not in uncut controls. Homozygous *ptpn11a^−/−^ ptpn11b^−/−^* zebrafish embryos expressed *mmp9* and *junbb* to an extent similar to that of their siblings following caudal fin fold amputation, suggesting that formation of the wound epidermis and the distal blastema occurred in Shp2-deficient zebrafish embryos.

### Arrested proliferation in zebrafish embryos deficient for Shp2 during regenerative outgrowth.

**FIG 4:**
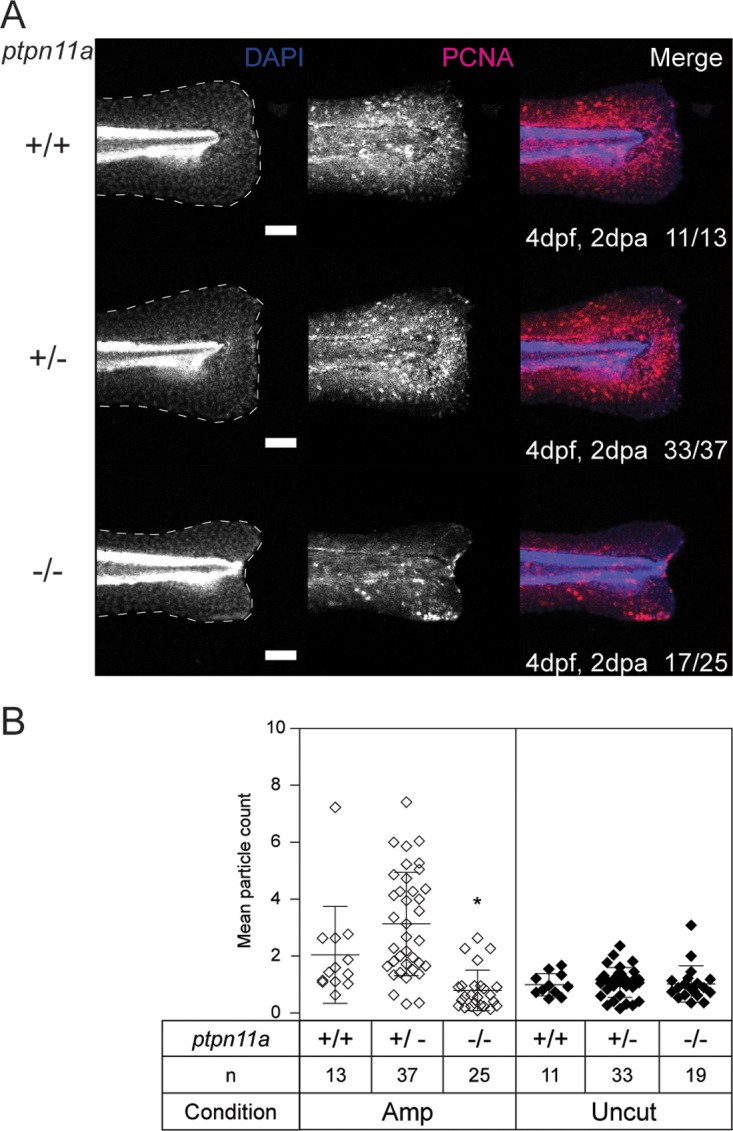
Proliferation is arrested at the amputated caudal fin fold margin of Shp2-deficient embryos. At 2 dpf, the caudal fin folds of embryos from a *ptpn11a^+/−^ ptpn11b^−/−^* incross were amputated and allowed to regenerate. The embryos were fixed at 2 dpa (4 dpf, 2 dpa) and subjected to whole-mount immunohistochemistry using an antibody specific for the cell proliferation marker PCNA (red). The embryos were counterstained with DAPI (4′,6-diamidino-2-phenylindole) (blue). Maximum-intensity projection images of the caudal fin folds were taken, and all the embryos were genotyped. (A) Representative images of amputated embryo caudal fin folds, with the edges of the fin folds indicated with dashed lines. The number of embryos showing similar patterns/total number of embryos analyzed are indicated in the bottom right corners of the images in the right-hand column. Scale bars, 100 μm. (B) PCNA immunofluorescence between the tip of the notochord and the edge of the caudal fin fold was quantified by mean particle count, with thresholding and size restriction to remove background signal. Equivalent uncut controls were also quantified, and the mean values of all the caudal fin folds are shown. The statistical significance of the means was determined relative to *ptpn11a^+/+^ ptpn11b^−/−^* zebrafish embryos within the amputated group, and likewise within the uncut group. *, *P* < 0.05; the error bars represent standard deviations.

During regenerative outgrowth, proliferation is upregulated to generate the cells required to form and replace the lost tissue (2, 3). We analyzed cell proliferation during the regenerative outgrowth stage by immunohistochemistry using an antibody specific for proliferating cell nuclear antigen (PCNA). Zebrafish embryos from a *ptpn11a^+/−^ ptpn11b^−/−^* incross were fixed at 2 dpa and subjected to whole-mount immunohistochemistry for detection of PCNA expression. All the zebrafish embryos were subsequently genotyped. At 2 dpa, PCNA immunofluorescence was dispersed and significantly reduced (*P* < 0.05) at the edges of the amputated caudal fin folds of *ptpn11a^−/−^ ptpn11b^−/−^* zebrafish embryos, whereas in siblings that regenerated normally, PCNA immunofluorescence was concentrated between the amputation plane and the wound margin (Fig. 4). PCNA immunofluorescence remained low in uncut controls (Fig. 4B). These results indicate that proliferation is reduced in *ptpn11a^−/−^ ptpn11b^−/−^* zebrafish embryos by 2 dpa compared to siblings in the regenerative outgrowth phase following caudal fin fold amputation.

### Reduced MAPK signaling in zebrafish embryos deficient for Shp2.

**FIG 5:**
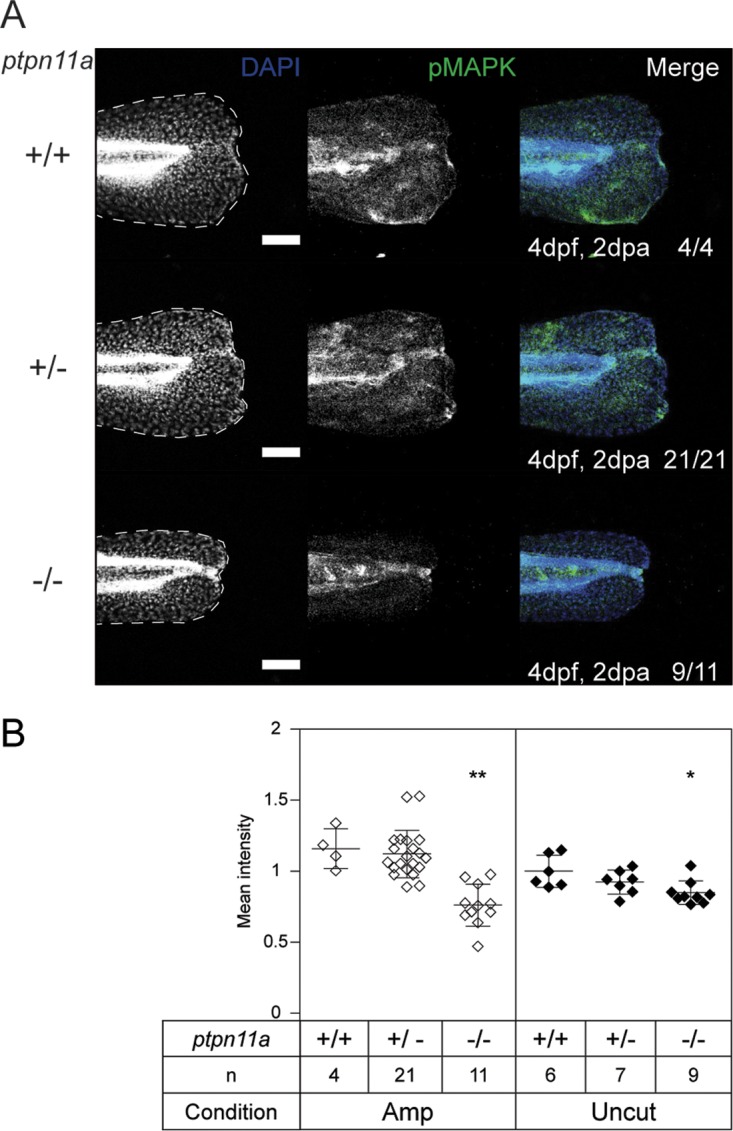
Reduced p-MAPK in regenerating caudal fin folds of Shp2-deficient embryos. At 2 dpf, the caudal fin folds of embryos from a *ptpn11a^+/−^ ptpn11b^−/−^* incross were amputated and allowed to regenerate. The embryos were fixed at 2 dpa (4 dpf, 2 dpa) and subjected to whole-mount immunohistochemistry using a p-MAPK-specific antibody (Thr202/Tyr204) (green). The embryos were counterstained with DAPI (blue). Maximum-intensity projection images were taken of the caudal fin folds, and all the embryos were genotyped. (A) Representative images of amputated embryo caudal fin folds, with the edges of the fin folds indicated with dashed lines. The number of embryos showing similar patterns/total number of embryos analyzed are indicated in the right-hand column. Scale bars, 100 μm. (B) p-MAPK was quantified by the mean intensity of the region between the notochord and the edge of the caudal fin fold. Equivalent uncut controls were also quantified, and the mean values of all the caudal fin folds are depicted. The statistical significance of the means was determined relative to *ptpn11a^+/+^ ptpn11b^−/−^* zebrafish embryos within the amputated group, and likewise within the uncut group. **, *P* < 0.01; *, *P* < 0.05; the error bars represent standard deviations.

Loss of SHP2 in tissue culture cells or in knockout mice results in reduced MAPK signaling, leading to reduced proliferation and differentiation and developmental defects (10, 17–19, 32, 61). Furthermore, activated MAPK signaling following injury is associated with regenerative competence across species (47, 48). To determine if loss of Shp2 affected MAPK signaling in *ptpn11a^−/−^ ptpn11b^−/−^* zebrafish embryos, we performed whole-mount immunohistochemistry using a phospho-MAPK-specific antibody (p-MAPK; phospho-p44/42 MAPK [Thr202/Tyr204]). In comparison to their siblings, *ptpn11a^−/−^ ptpn11b^−/−^* zebrafish embryos displayed significantly reduced p-MAPK levels following caudal fin fold amputation at 4 dpf (*P* < 0.01) (Fig. 5A). Note that, compared to their siblings, p-MAPK levels were also significantly reduced in the caudal fin folds of uncut *ptpn11a^−/−^ ptpn11b^−/−^* zebrafish embryos (*P* < 0.05) (Fig. 5B). These immunohistochemistry experiments indicated that MAPK signaling is reduced in zebrafish embryos lacking functional Shp2.

### Reducing MAPK signaling by pharmacological inhibition of MEK1 phenocopies loss of Shp2 in zebrafish caudal fin fold regeneration.

**FIG 6:**
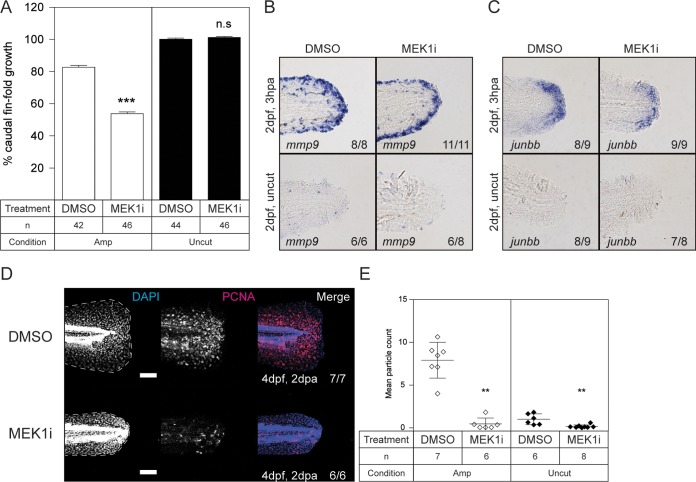
Impaired caudal fin fold regeneration in wild-type zebrafish embryos treated with MEK1 inhibitor. At 2 dpf, the caudal fin folds of wild-type embryos were amputated and allowed to regenerate in the presence of 50 nM PD184352 (MEK1i) or 1% DMSO (solvent control). (A) Regeneration after 3 days was quantified by measuring the distance from the tip of the notochord to the edge of the caudal fin fold. By 3 dpa, regeneration of the caudal fin fold of control zebrafish embryos was 80% complete. The means of caudal fin fold growth are depicted relative to caudal fin fold growth of DMSO-treated uncut controls. The statistical significance of the mean of PD184352 (MEK1i)-treated amputated embryos was determined relative to the mean of DMSO-treated amputated embryos, and likewise for the uncut treated and untreated embryos. The number of embryos is indicated (*n*). ***, *P* < 0.001; n.s. not significant; the error bars indicate standard errors of the mean. (B and C) Embryos were fixed at 3 hpa, or the equivalent for uncut controls, and subjected to hybridization for *mmp9* (B) or *junbb* (C). Representative images of caudal fin folds of embryos are shown, and the number of embryos showing similar patterns/total number of embryos analyzed are indicated in the bottom right corner of each image. (D) Embryos were fixed at 2 dpa (4 dpf, 2 dpa) and subjected to whole-mount immunohistochemistry using an antibody specific for the cell proliferation marker PCNA (red). The embryos were counterstained with DAPI (blue). Maximum-intensity projection images were taken of the caudal fin folds. Representative images of amputated embryo caudal fin folds are shown, with the edges of the fin folds indicated with dashed lines. The number of embryos showing similar patterns/total number of embryos analyzed are indicated in the right-hand column. Scale bars, 100 μm. (E) PCNA immunofluorescence between the tip of the notochord and edge of the caudal fin fold was quantified by mean particle count, with thresholding and size restriction to remove background signal. Equivalent uncut controls were also quantified. The means of the amputated PD184352 (MEK1i)-treated group were compared to those of the amputated DMSO-treated group, and likewise, the means of the uncut PD184352 (MEK1i)-treated group were compared to those of the uncut DMSO-treated group. **, *P* < 0.01; the error bars represent standard deviations.

We hypothesized that the reduced MAPK signaling observed in Shp2-deficient zebrafish embryos was responsible for the lack of caudal fin fold regeneration. We therefore tested if pharmacological inhibition of MAPK signaling in zebrafish embryos impaired caudal fin fold regeneration. The caudal fin folds of wild-type zebrafish embryos (*ptpn11a^+/+^ ptpn11b^+/+^*) were amputated at 2 dpf and allowed to regenerate for 3 days in the presence of 50 nM the MEK1 inhibitor PD184352 (also known as CI-1040) or solvent (1% dimethyl sulfoxide [DMSO]) as a control. Treatment of zebrafish embryos with PD184352 significantly impaired caudal fin fold regeneration (*P* < 0.001) (Fig. 6A). Uncut control zebrafish embryos showed that PD184352 treatment by itself did not affect normal caudal fin fold growth (Fig. 6A).

We investigated if the impaired regeneration of wild-type zebrafish embryos treated with PD184352 was associated with defective wound healing or distal blastema formation. Wild-type zebrafish embryos were amputated at 2 dpf and treated with PD184352 or DMSO until fixation at 3 hpa. Equivalent uncut controls were treated and fixed. Zebrafish embryos were subjected to *in situ* hybridization using an *mmp9*-specific or *junbb*-specific probe for detection of the wound epidermis and distal blastema, respectively. Expression of *mmp9* (Fig. 6B) and *junbb* (Fig. 6C) was clearly induced in amputated caudal fin folds but not in uncut control zebrafish embryos. PD184352-treated zebrafish embryos expressed *mmp9* and *junbb* to an extent similar to that of solvent-treated control embryos following caudal fin fold amputation, suggesting that formation of the wound epidermis and subsequent formation of the distal blastema were not affected by PD184352-mediated inhibition of MAPK signaling.

Next, we analyzed cell proliferation during the regenerative outgrowth stage of zebrafish embryos treated with PD184352. Wild-type zebrafish embryos had their caudal fin folds amputated at 2 dpf and were treated with PD184352 or solvent until fixation at 2 dpa. The zebrafish embryos were subjected to whole-mount immunohistochemistry for detection of PCNA expression. At 2 dpa, PCNA immunofluorescence in amputated caudal fin folds was dispersed and significantly reduced (*P* < 0.01) in zebrafish embryos treated with PD184352 compared to control zebrafish embryos treated with DMSO (Fig. 6D). Baseline PCNA staining in the caudal fin folds of uncut control zebrafish embryos at 4 dpf was low but was also significantly reduced following PD184352 treatment (*P* < 0.01) (Fig. 6E). These results demonstrate that MAPK signaling is required for normal caudal fin fold regeneration of zebrafish embryos and promotes proliferation during regenerative outgrowth.

## DISCUSSION

Our results demonstrate a critical role for Shp2 and MAPK signaling in zebrafish embryo caudal fin fold regeneration. Zebrafish embryos lacking functional Shp2 (*ptpn11a^−/−^ ptpn11b^−/−^*) show severely impaired regeneration of their caudal fin folds following amputation (Fig. 1). Expression of wild-type Shp2a rescues regeneration, which relies on its SH2 domains and catalytic activity (Fig. 1). The initial response to amputation includes formation of the wound epidermis and distal blastema, which are characterized by increased expression of *mmp9* and *junbb*, respectively, and our *in situ* hybridization results suggest that these two processes do occur in Shp2-deficient zebrafish embryos (Fig. 3). Critically, immunohistochemistry for PCNA revealed that proliferation was arrested during the regenerative outgrowth phase in Shp2-deficient zebrafish embryos (Fig. 4). We propose that the reduced p-MAPK levels in *ptpn11a^−/−^ ptpn11b^−/−^* zebrafish embryos (Fig. 5) cause impaired caudal fin fold regeneration, which is consistent with our observation that MEK1 inhibition phenocopies loss of Shp2 in zebrafish embryo caudal fin fold regeneration, impairing zebrafish caudal fin fold regeneration and reducing proliferation during regenerative outgrowth (Fig. 6).

Recently, we demonstrated that Shp2a and Shp2b are two of the eight PTPs that are oxidized and hence inactivated in response to caudal fin amputation (54). Here, we demonstrate that Shp2 signaling is required for regeneration of the zebrafish embryo caudal fin fold, which seems to contrast with the finding that Shp2 is oxidized and thus inactivated upon amputation of the zebrafish caudal fin. However, production of reactive oxygen species (ROS) in response to caudal fin amputation is transient (62). Presumably, Shp2 is transiently inactivated by the production of ROS following zebrafish caudal fin amputation, and Shp2 is subsequently reduced again to an active form that is required for caudal fin regeneration. Whether transient inactivation of Shp2 is required for caudal fin regeneration remains to be determined.

SHP2 has an important signaling role in many cellular processes (35, 63) and interacts with associated proteins and substrates through its SH2 domains and/or C-terminal domain (25, 41, 42). Our rescue experiments indicated that the SH2 domains, but not the tyrosine phosphorylation sites in the C-terminal domain of Shp2a, are required to rescue caudal fin fold regeneration of *ptpn11a^−/−^ ptpn11b^−/−^* zebrafish embryos (Fig. 1). Mutation of the two SH2 domains impairs the association of SHP2 with phosphorylated growth factor receptors and substrates (64) and has been shown to inhibit EGF stimulation of MAPK activation in cells (65). Thus, the inability of Shp2a-R32M-R138M to rescue caudal fin fold regeneration suggests that Shp2a binding to substrates or interacting proteins via its SH2 domains is required. Mutating Y542 and Y580 prevents binding of SHP2 to GRB2 and reduces, but importantly does not abolish, the activation of MAPK in response to stimulation by some growth factors in tissue culture cells, suggesting that the SHP2-GRB2 interaction is dispensable in some contexts (11, 41, 66). We conclude that expression of Shp2a-Y542F-Y580F in *ptpn11a^−/−^ ptpn11b^−/−^* zebrafish embryos apparently mediated sufficient MAPK activation to rescue caudal fin fold regeneration.

The catalytic activity of SHP2 is paramount for regulation of MAPK signaling (11). We provide evidence that Shp2a-R466M, which lacks detectable catalytic activity, fails to rescue caudal fin fold regeneration in *ptpn11a^−/−^ ptpn11b^−/−^* zebrafish embryos (Fig. 1), yet Shp2a-A462T, which harbors very low, but detectable, catalytic activity did rescue regeneration. Shp2a-D61G, with enhanced catalytic activity compared to wild-type Shp2a, rescued regeneration in *ptpn11a^−/−^ ptpn11b^−/−^* zebrafish embryos to an extent similar to that of Shp2a-A462T (Fig. 1B). These results are surprising, particularly because tight regulation of signal transduction has been demonstrated to be essential for zebrafish caudal fin regeneration (45, 67, 68). It is not unlikely that differences in the conformation dynamics of Shp2a mutants affect the function of Shp2a. The current model for SHP2 is that under control conditions, it is in the closed conformation, through interactions between the SH2 domains and the PTP domain (30). Ligation of the SH2 domains to phosphotyrosine residues on other proteins prompts an open conformation, allowing the PTP domain to dephosphorylate substrates. “Activating” mutations, such as D61G in the N-terminal SH2 domain, disrupt the interaction between the SH2 domains and the PTP domain and stabilize the open conformation of SHP2 (36–38). Recently, it has been hypothesized that while the SHP2 A461T mutant has reduced catalytic activity, it is also stabilized in an open conformation, allowing prolonged association with substrates that compensates for its reduced activity (39, 69). This would explain why both Shp2a-D61G and Shp2a-A462T rescue zebrafish embryo caudal fin fold regeneration. It would be interesting to investigate the effects of Shp2a-D61G and Shp2a-A462T on downstream signaling during caudal fin fold regeneration.

The expression of *mmp9* and *junbb* has been shown to be specifically increased in the wound epidermis and distal blastema, respectively, following zebrafish embryo caudal fin fold amputation (59). Furthermore, *junbb* expression is maintained well into the initial stage of regenerative outgrowth (60), indicating that *junbb* is a definitive distal blastema marker. We show that the amputated caudal fin folds of *ptpn11a^−/−^ ptpn11b^−/−^* zebrafish embryos express *mmp9* and *junbb*, like those of their siblings (Fig. 3), suggesting that both wound healing and distal blastema formation occur in the absence of Shp2. This appears to contrast with previous results showing that Fgfr1 signaling is required for blastema formation (50, 68). Whereas there are overlaps in FGFR1 and SHP2 signaling (52), apparently, Fgfr1 and Shp2a signaling in zebrafish differ to such an extent that distal blastema formation is dependent on Fgfr1 but not on Shp2.

The next stage of regeneration, regenerative outgrowth, is characterized by proliferation and differentiation of cells to replace the lost tissue. Our whole-mount immunohistochemistry experiments demonstrated that cell proliferation and MAPK phosphorylation are significantly reduced in the regenerating caudal fin folds of *ptpn11a^−/−^ ptpn11b^−/−^* zebrafish embryos compared to their siblings (Fig. 4 and 5). Inhibiting MAPK signaling using an inhibitor of MEK1 was sufficient to phenocopy the effect of loss of Shp2 (Fig. 6). Our results using the MEK1 inhibitor endorse the conclusion that MAPK signaling is required to drive proliferation during regenerative outgrowth of zebrafish embryo caudal fin fold regeneration. These results are consistent with previous work demonstrating a requirement for Fgfr1 signaling in proliferation during zebrafish caudal fin regeneration (49, 51, 68) and reduced proliferation and regeneration in response to MEK1 inhibition during zebrafish heart regeneration (70). Considering this, Shp2a-MAPK signaling may have a conserved role in the regeneration of various tissues.

Collectively, our results suggest an essential role for Shp2a-mediated MAPK signaling in promoting cell proliferation during the regenerative outgrowth phase of regenerating zebrafish embryo caudal fin folds. Recent work has shown that loss of SHP2 in mice, resulting in reduced MAPK signaling and reduced proliferation, leads to impaired muscle regeneration, and this was attributed to satellite cell quiescence (71). Possibly, the loss of Shp2 in zebrafish embryo caudal fin folds induces quiescence in dedifferentiated cells of the distal blastema. This would certainly be in concordance with our results showing that regenerative outgrowth was impaired, despite apparently normal distal blastema formation. In addition to promoting MAPK signaling, SHP2 has been shown to promote or inhibit PI3K signaling (72, 73). Interestingly, the symptoms that present *in vivo* as a result of loss of *ptpn11* or activating mutations of SHP2 appear to be primarily due to the effect on MAPK signaling. For example, mice expressing the activating SHP2 mutant Q79R display MAPK hyperphosphorylation and congenital heart defects, while both these phenotypes are ameliorated in Q79R × *Erk1^−/−^* mice (74). In comparison, genetic ablation of PTPN11 in retinal cells results in reduced MAPK phosphorylation but does not affect AKT phosphorylation (61). The defects that result are also not rescued by mutating the antagonist of PI3K signaling, phosphatase, and tensin homolog (PTEN), which normally increases PI3K signaling (75). However, hyperactive KRas, which has been shown to alleviate the requirement for SHP2 in the maintenance of hematopoietic stem cells (17), does rescue the retinal defects. As PI3K signaling has previously been shown to be required for blastema formation (45) and our results suggest that distal blastema formation occurs in zebrafish embryos lacking functional Shp2, we conclude that it is unlikely that Shp2 acts through PI3K signaling during zebrafish embryo caudal fin fold regeneration.

In conclusion, we have demonstrated that Shp2a signaling is indispensable for zebrafish embryo caudal fin fold regeneration. Our results are consistent with Shp2a acting to promote MAPK signaling, thus coordinating proper proliferation during regenerative outgrowth of the zebrafish embryo caudal fin fold.

## MATERIALS AND METHODS

### Zebrafish husbandry.

All procedures involving experimental animals were performed under license number GZB/VVB 2041019 of the Hubrecht Institute/Royal Academy of Arts and Sciences (Koninklijke Nederlandse Akademie van Wetenschappen [KNAW]) and approved by the local animal experiment committee according to local guidelines and policies in compliance with national and European laws.

The *ptpn11a^+/−^ ptpn11b^−/−^* zebrafish lines in the Tuebingen long fin (TL) background were previously created by target-selected gene inactivation (TSGI), and the both *ptpn11a^hu3459^* and *ptpn11b^hu5920^* alleles result from nonsense mutations that lead to a premature stop codon upstream of the catalytic cysteine, C460 (26). Adult *ptpn11a^+/−^ ptpn11b^−/−^* zebrafish were incrossed to generate *ptpn11a^−/−^ ptpn11b^−/−^* zebrafish embryos for all experiments. The zebrafish were raised and maintained as described by Westerfield (56) under a 14-h light/10-h dark cycle at 28.5°C.

### mRNA synthesis and microinjections.

All constructs contained an N-terminal eGFP connected by a peptide-2a cleavage sequence (55). The constructs pCS2^+^-eGFP-2a-Shp2a (WT), pCS2^+^-eGFP-2a-Shp2a-R466M, pCS2^+^-eGFP-2a-Shp2a-R32M-R138M, pCS2^+^-eGFP-2a-Shp2a-Y542F-Y580F, pCS2^+^-eGFP-2a-ptpn11a-D61G, and pCS2^+^-eGFP-2a-ptpn11a-A462T were obtained as described previously (76). Sense mRNA synthesis and microinjection into one-cell stage zebrafish embryos were performed as described previously (77).

### Caudal fin fold amputation.

Zebrafish embryo amputations were performed as previously described (78) at 2 dpf for all experiments. Regeneration was allowed to proceed until analysis at 3 dpa or fixation at 3 hpa or 2 dpa. PD184352 (Sigma) or dimethyl sulfoxide (Sigma) was administered directly following recovery of amputated zebrafish embryos in E3 medium (5 mM NaCl, 0.17 mM KCl, 0.33 mM CaCl_2_, 0.33 mM MgSO_4_). Whole zebrafish embryos were lysed for genotyping or fixed in 4% paraformaldehyde (PFA) in phosphate-buffered saline (PBS), either 3 hpa for *in situ* hybridization or at 2 dpa for immunohistochemistry.

### *In situ* hybridization.

*In situ* hybridizations were performed as previously described (79), using *mmp9* or *junbb* digoxigenin-UTP-labeled antisense riboprobes. Parts of *mmp9* and *junbb* were amplified from zebrafish cDNA using specific primers (Table 1), and the resulting products from the nested PCR were used as DNA templates for synthesis of digoxigenin-UTP-labeled antisense riboprobes. Following staining, the caudal fin folds of zebrafish embryos were severed and mounted in 70% glycerol in PBS for imaging on a Zeiss Axioskop 2 Mot Plus microscope with a Plan-Neofluar 10×/0.30-numerical-aperture or 20×/0.50-numerical-aperture objective. The rest of the zebrafish embryo was lysed for genotyping.

**TABLE 1:** Primers used for amplifying *mmp9* and *junbb* from zebrafish embryo cDNA and *ptpn11a* from genomic zebrafish DNA for genotyping

Primer	Sequence
*mmp9* FWD 1	TCCTGGAGATGTGATCAAGAA
*mmp9* REV 1	GGCCCTCACTGGTGCAGGATG
*mmp9* FWD 2	CACAGCTAGCGGATGAGTATCTGAAGC
*mmp9*-T7 REV 2	TAATACGACTCACTATAGAATGGAAAATGGCATGGCTCTCC
*junbb* FWD 1	TGGGTTACGGTCACAACGAC
*junbb* REV 1	CAGTGTCCGTTCTCTTCCGT
BamHI-*junbb* FWD (nested)	ATAGGATCCTACACGACGCTGAACGCATA
*junbb*-EcoRI REV (nested)	CTCGAATTCGTGTCCGTTCTCTTCCGTCC
*junbb* FWD 2	TACACGACGCTGAACGCATA
*junbb*-T7 REV 2	TAATACGACTCACTATAGGTGTCCGTTCTCTTCCGTCC
*Ptpn11a* WT FWD	GAAGGTGACCAAGTTCATGCTACATGGAGCATCATGGAC
*Ptpn11a* KO FWD	GAAGGTCGGAGTCAACGGATTACATGGAGCATCATGGAT
*Ptpn11a* REV	AGCTCAATGACGTCTCCGTTTTTCTCTTT
*Ptpn11a* FWD 1	GCGCTGTCACACACATTAAGA
*Ptpn11a* REV 2	TCACAGCCAATAAAGAGAAGC
*Ptpn11a* FWD (nested) 3	CGACCTGTATGGTGGAGAGAA
*Ptpn11a* REV (nested) 4	TCCCAAATTGTCATGTAAGG

### Immunohistochemistry.

Zebrafish embryos fixed in 4% PFA were washed in PBS-0.1% Tween 20, and antigen retrieval was performed depending on the antibody used: ice-cold acetone for 20 min for PCNA and 10 mM Tris, 1 mM EDTA, pH 9.0, for phospho-p44/42 MAPK (Thr202/Tyr204). Whole zebrafish embryos were incubated overnight at 4°C in mouse anti-PCNA (1:200; number M0879; Dako Agilent Pathology Solutions), or rabbit anti-phospho-p44/42 MAPK (Thr202/Tyr204) (1:100; number 4370; Cell Signaling Technology). Secondary antibodies conjugated to Cy5 goat anti-mouse or goat anti-rabbit IgG (number 115-175-146 and number 111-175-144; Jackson ImmunoResearch) were used at 1:500 and 1:200, respectively. Nuclei were shown by DAPI (4′,6-diamidino-2-phenylindole) staining. Caudal fin folds of zebrafish embryos were mounted for imaging in 70% glycerol in PBS, and the rest of the zebrafish embryo was lysed for genotyping. Z-stacks (6-μm step size) of the caudal fin fold were acquired for every zebrafish embryo with a Leica Sp8 confocal microscope. ImageJ software (http://rsb.info.nih.gov./ij/) was used to generate maximum-intensity (z) projections and merge channels. Quantification was performed on the original z-projections (1,024 by 1,024 pixels [px]; 300 dots/in [dpi]). PCNA immunostaining between the tip of the notochord and the edge of the caudal fin fold of zebrafish embryos from a *ptpn11a^+/−^ ptpn11b^−/−^* incross were quantified by cropping the z-projections to remove the signal adjacent to the tip of the notochord and applying rolling-ball background subtraction with an average of 15 px. Particles were counted for PCNA immunofluorescence following black-and-white thresholding of 40 to 255, applying watershed, and using a size restriction of 0.00009 in^2^ to infinity. PCNA immunostaining between the tip of the notochord and the edge of the caudal fin fold of wild-type zebrafish embryos treated with PD184532 or DMSO was quantified in an identical manner, applying rolling-ball background subtraction with an average of 100 px. Immunostaining of p-MAPK was performed following rolling-ball background subtraction with an average of 50 px. The mean intensity of p-MAPK was measured from the wound margin inward using a region of interest with dimensions equivalent to the following: height (h), 2.08 μm (625 px), and various widths (w) for each sample, i.e., for embryos at 2 dpa, 0.82 μm (246 px) for amputated zebrafish embryos and 1.00 μm (300 px) for uncut zebrafish embryos.

### Genotyping.

All the zebrafish embryos that were used in these assays were genotyped to establish their *ptpn11a* status. To this end, genomic zebrafish DNA was extracted through lysis of the zebrafish embryos in 100 μg/ml proteinase K (Sigma) diluted in SZL buffer (50 mM KCl, 2.5 mM MgCl, 10 mM Tris, pH 8.3, 0.005% NP-40, 0.005% Tween 20, and 10% 0.1% gelatin). Lysis was performed by incubating at 60°C for 1 h, followed by 95°C for 15 min in a thermal cycler (Bio-Rad T100). The *ptpn11a^hu1864^* allele in nonfixed tissue was analyzed by Kompetitive allele-specific PCR (KASP): primers of *ptpn11a* containing nonsense mutations of the *ptpn11a^hu1864^* allele (Table 1) were mixed with genomic zebrafish DNA and KASP master mix (LGC Group). Amplification was carried out according to the manufacturer's instructions, and the resulting PCR products were analyzed in a Pherastar microplate reader (BMG Labtech). Klustercaller software (LGC Group) was used to identify the mutations. For fixed tissue, genotyping for the *ptpn11a^hu1864^* allele was performed by nested PCR with primer sets 1 to 4 (Table 1), followed by Sanger sequencing (Macrogen Inc., Europe) to detect the mutations.

### Immunoblotting.

Whole-cell protein extracts from human embryonic 293 T cells transfected with and overexpressing zebrafish *ptpn11a* were prepared by lysis in ice-cold buffer (25 mM HEPES, pH 7.4, 150 mM NaCl, 0.25% deoxycholate, 1% Triton X-100, 10 mM MgCl_2_, 1 mM EDTA, and 10% glycerol) containing protease and phosphatase inhibitors. Samples were centrifuged at 12,000 × *g* for 20 min, and the supernatant was collected. Proteins were resolved in 10% SDS-PAGE gels under reducing conditions and transferred to polyvinylidene difluoride (PVDF) membranes (IPVH00010; Merck Millipore). Immunoblotting was performed using anti-SHP2 (SH-PTP2 C-18; number SC-280; Santa Cruz Technology) or antitubulin (number CP06; Merck Millipore) antibody, followed by horseradish peroxidase (HRP)-conjugated anti-rabbit or anti-mouse antibodies, respectively. Detection was done by enhanced chemiluminescence (number 34095; Thermo Fischer Scientific).

### Statistics.

For analysis of caudal fin fold lengths, histograms of whole data sets were examined to determine nonnormal distribution of the data. Statistical analysis of unequal variances was obtained through a Kruskall-Wallis test. Differences between different experimental conditions were assessed for significance using a Mann-Whitney U test. Differences were considered significant at a *P* value of <0.001 and if they satisfied a confidence interval of 95% in a Monte Carlo exact test. All tests for regenerating caudal fin folds were performed in SPSS (IBM). For analysis of immunohistochemistry measurements, differences between different experimental conditions were assessed for significance using a Mann-Whitney U test with a confidence level set to 95%. All tests for immunohistochemistry measurements were performed in GraphPad Prism (GraphPad Software). Differences were considered significant at a *P* value of <0.05.
